# Activated Wnt signaling induces myofibroblast differentiation of mesenchymal stem cells, contributing to pulmonary fibrosis

**DOI:** 10.3892/ijmm.2014.1672

**Published:** 2014-02-25

**Authors:** ZHAORUI SUN, CONG WANG, CHAOWEN SHI, FANGFANG SUN, XIAOMENG XU, WEIPING QIAN, SHINAN NIE, XIAODONG HAN

**Affiliations:** 1 Immunology and Reproductive Biology Laboratory, Medical School of Nanjing University, Nanjing, Jiangsu 210093, P.R. China; 2 Jiangsu Key Laboratory of Molecular Medicine, Nanjing University, Nanjing, Jiangsu 210093, P.R. China; 3 State Key Laboratory of Analytical Chemistry for Life Science, Nanjing University, Nanjing, Jiangsu 210093, P.R. China; 4 State Key Laboratory of Bioelectronics, Southeast University, Nanjing, Jiangsu 210093, P.R. China; 5 Department of Emergency, Jinling Hospital, Medical School of Nanjing University, Nanjing, Jiangsu 210002, P.R. China

**Keywords:** lung injury, mesenchymal stem cells, pulmonary fibrosis, stromal cell-derived factor-1/CXC chemokine receptor 4, Wnt/β-catenin signaling

## Abstract

Acute lung injury may lead to fibrogenesis. However, no treatment is currently available. This study was conducted to determine the effects of bone marrow-derived mesenchymal stem cells (MSCs) in a model of HCl-induced acute lung injury in Sprague-Dawley (SD) rats. Stromal cell-derived factor (SDF)-1 and its receptor CXC chemokine receptor (CXCR)4 have been shown to participate in mobilizing MSCs. Adenovirus carrying the CXCR4 gene was used to transfect MSCs in order to increase the engraftment numbers of MSCs at injured sites. Histological examination data demonstrated that the engraftment of MSCs did not attenuate lung injury and pulmonary fibrosis. The results showed that engraftment of MSCs almost differentiated into myofibroblasts, but rarely differentiated into lung epithelial cells. Additionally, it was demonstrated that activated canonical Wnt/β-catenin signaling in injured lung tissue regulated the myofibroblast differentiation of MSCs *in vivo*. The *in vitro* study results demonstrated that activation of the Wnt/β-catenin signaling stimulated MSCs to express myofibroblast markers; however, this process was attenuated by Wnt antagonist DKK1. Therefore, the results demonstrated that the aberrant activation of Wnt signaling induces the myofibroblast differentiation of engrafted MSCs, thus contributing to pulmonary fibrosis following lung injury.

## Introduction

Acute lung injury (ALI) is a clinical syndrome defined by acute hypoxemic respiratory failure and bilateral pulmonary infiltrates consistent with edema, eventually leading to lung fibrogenesis (1). ALI is considered an immutable response to lung injury with transition from alveolar capillary damage to a fibroproliferative phase, independent of initial cause (2,3). Currently, no specific strategies for the treatment of ALI are available. It has been previously suggested that bone marrow-derived mesenchymal stem cells (MSCs) incorporate into various tissues and in some cases differentiate into a variety of cell types of the tissue to which they have homed (4–8). Conceivably, MSCs engrafted into injured lung tissue may assume lung tissue cell type and contribute to lung repair. This fact provides a strong rationale for exploring the potential use of MSCs for the treatment of ALI.

Migration of circulating MSCs to the injured sites is regulated by chemotactic signals. Stromal cell-derived factor (SDF)-1 and its receptor CXC chemokine receptor (CXCR)4 have been demonstrated to be the critical chemokines determining homing and engraftment of MSCs associated with injury repair in many tissue types (9–11). SDF-1 expression is upregulated in the injured tissue sites (12). CXCR4 is a critical chemokine receptor in stem cell homing, and the differential expression of SDF-1 in injured tissue creates a gradient essential for the migration of CXCR4-expressing cells. The SDF-1/CXCR4 chemotactic axis is crucial for recruitment of circulating or intravenously infused cells, suggesting that modulation of these interactions may enhance stem cell engraftment following injury (13–15). Therefore, the SDF-1/CXCR4 chemotactic axis was used to enhance homing and engraftment of MSCs by transfecting with adenovirus combined with CXCR4 gene and the therapeutic effect of MSCs for ALI was investigated.

MSCs localize to injured lungs and differentiate into specific lung cell types, including alveolar epithelial cells under appropriate conditions in order to promote lung remodeling. However, it was shown that bone marrow-derived circulating progenitor cells including bone marrow-derived MSCs accumulate in the lung and differentiate into lung fibroblasts, lung myofibroblasts and interstitial monocytes that contribute to a fibrotic response (16–18). Xu *et al* (18) clarified that lung injury induces the recruitment of CXCR4^+^ bone marrow-derived MSCs which may assume a fibroblast phenotype and contribute to fibrogenesis in the fibrogenic environment of the injured lung (18). The origin and nature of the factors or signaling pathways that determine the differentiation of MSCs remains to be determined, and the mechanisms of stem cell involvement in tissue repair, regeneration and remodeling remain unclear. It is therefore necessary to explore the possible mechanisms that may regulate the differentiation of engraftment MSCs *in vivo*. The types of cells differentiated from MSCs are important for lung repair or pulmonary fibrosis.

Wnt/β-catenin signaling plays an important role in tissue repair, wound closure, fibrosis and tissue remodeling (19–21). Previously it was demonstrated that aberrant activation of Wnt/β-catenin signaling has been connected with pathogenesis of lung disease, such as asthma, pulmonary fibrosis and lung cancer (22,23). Wnt/β-catenin signaling also plays a vital role in fate determination and differentiation of MSCs (24). The results of those studies suggested that Wnt/β-catenin signaling may participate in lung repair or pulmonary fibrosis via regulation of MSC differentiation into lung tissue-specific cells.

In the present study, we investigated the effect of intravenous transplantation of MSCs using the SDF-1/CXCR4 chemotactic axis to increase engraftment numbers in order to modify the pathophysiology of HCl-induced lung injury. Additionally, the role of the Wnt/β-catenin signaling pathway in the regulation of MSC differentiation *in vivo* was examined.

## Materials and methods

### Ethics statement

The rats received care in compliance with the Guide for the Care and Use of Experimental Animals formulated by the National Society for Medical Research. the study was approved by Nanjing University.

### MSC isolation, culture and flow cytometry

Adult Sprague-Dawley (SD) rats were purchased from the Animal Feeding Center of Nanjing Medical University. MSC isolation, culture and flow cytomery were performed as previously described (24). In brief, MSCs were obtained from the bone marrow of the femurs and tibias of rats weighing 70–80 g. The marrow was flushed out using PBS under aseptic conditions. The collected cells were filtered through a 70 μm cell strainer and centrifuged at 300 × g for 8 min. The cells were subsequently cultured in low-glucose Dulbecco’s modified Eagle’s medium (DMEM; HyClone, Thermo Scientific, San Jose, CA, USA) with 10% FBS (Gibco-BRL, Invitrogen Life Technologies, Paisley, UK; Invitrogen Life Technologies, Carlsbad, CA, USA), 1% L-glutamine, and a 1% solution of penicillin and streptomycin seeded at a density of 1×10^6^ cells/ml into uncoated flasks, and cultured in a humidified incubator at 37ºC in 5% CO_2_. Non-adherent cells were removed after 72 h. When MSCs reached 80% confluence, they were routinely passaged using 0.25% trypsin, with a dilution of 1:2 at each passage.

For flow cytometry, 1×10^5^ passage-3 MSCs were incubated with fluorescence-conjugated primary antibodies at 37ºC for 1 h in the dark following two washes with PBS and then incubated with secondary antibody at 37ºC for 30 min. After washing three times with PBS, the cells were analyzed using a FACSCalibur flow cytometer and analyzed with Paint-A-Gate software (Becton-Dickinson, San Jose, CA, USA). The antibodies employed were: PE anti-rat CD29 from BioLegend, Inc. (San Diego, CA, USA), R-PE anti-rat CD44 from Antigenix America, Inc. (Melville, NY, USA), PE anti-rat CD90 from eBioscience, Inc. (San Diego, CA, USA), mouse anti-rat CD73 from BD Pharmingen, Inc. (Franklin Lakes, NJ, USA), mouse anti-rat CD34 from Santa Cruz Biotechnology, Inc. (Santa Cruz, CA, USA), rabbit anti-rat CD133 and FITC anti-rat CD79 from Abcam, Inc. (Cambridge, MA, USA), FITC anti-rat CD11B and FITC anti-rat CD45 from Millipore Corporation (Billerica, MA, USA).

### Transfection of MSCs and immunofluorescent staining

The recombinant adenovirus vectors carrying the GFP reporter gene (Ad-GFP) or CXCR4-GFP (Ad-CXCR4-GFP) were obtained from Cyagen Biosciences, Inc. (Guangzhou, China). For transfection, passage-3 MSCs were seeded at 1×10^4^ cells/cm^2^ in a T-75 cm^2^ flask. The following day, MSCs were transfected with Ad-GFP or Ad-CXCR4-GFP in FBS-free DMEM medium at a multiplicity of infection (MOI) of 100 for 16 h. After transfection, DMEM containing adenoviral particles was removed and fresh MSC culture medium was added. Forty-eight hours after transfection, MSCs were prepared for injection, and western blot assay and immunofluorescence analysis were performed.

Immunofluorescent analysis of MSC-Ad-CXCR4/GFP cells was performed as previously described (25). Briefly, MSCs transfected with Ad-GFP or Ad-CXCR4-GFP for 48 h were first fixed with 4% paraformaldehyde for 10 min. To block non-specific binding sites, the cells were incubated with PBS containing 2% bovine serum albumin (BSA) for 1 h at 37ºC. The primary antibody CXCR4 (Abcam, Inc.) was diluted at a certain concentration (10 μg/ml) according to the manufacturer’s instructions. Incubation was performed at 4ºC for 16 h. After three washes with PBS, MSCs were incubated with a secondary antibody (goat anti-rabbit Alexa Fluor 594; Invitrogen Life Technologies, Gaithersburg, MD, USA) at a 1:400 dilution in 2% BSA for 1 h at 37ºC in the dark. Non-transfection MSCs were used as the control. The cells were stained with 5 μg/ml 4′,6′-diamidino-2-phenylindole (DAPI) (Biyuntian, Inc., Nantong, Jiangsu, China) to identify cellular nuclei. Images were captured using a confocal fluorescence microscope (Olympus, Tokyo, Japan).

### Hydrochloric acid-induced lung injury and MSC administration

Male 8- to 10-week-old SD rats were anesthetized via an intraperitoneal injection of ketamine (60 mg/kg) and diazepam (60 mg/kg), then hydrochloric acid (HCl, pH 1.5, 1 ml/kg) was administered intranasally. Normal controls were administered with PBS (1 ml/kg) in the lung. MSC-Ad-GFP/CXCR4 cells (5×10^6^ cells in 200 μl of PBS) were administered via tail vein injection to rats 24 h after lung injury.

### Animal groups and study design

SD rats were randomly divided into four groups (n=24 for each group): control: normal controls were injected with MSC-Ad-CXCR4 cells. ALI: HCl instillation and injection of PBS; ALI+MSC-GFP: ALI rats were injected with MSC-Ad-GFP cells; and ALI+MSC-CXCR4: ALI rats were injected with MSC-Ad-CXCR4 cells.

### Reverse transcription-polymerase chain reaction (RT-PCR)

Semiquantitative RT-PCR was performed as previously described (26). CXCR4 mRNA was obtained from MSC-Ad-GFP and MSC-Ad-CXCR4 cells. SDF-1 and immunoreactive cytokine [tumor necrosis factor-α (TNF-α), IL-6, IL-1β, IL-4, transforming growth factor-β1 (TGF-β1) and IL-10] mRNA were obtained from lung tissue following HCl administration at 1, 3, 7 and 14 days. Total RNA was extracted using TRIzol (Invitrogen Life Technologies, Gaithersburg, MD, USA) according to the manufacturer’s instructions. cDNA was generated from 2 μg of total RNA using random primers and EasyScript First-Strand cDNA Synthesis SuperMix. RT-PCR was performed using primers specific for the factors of interest. Quantification of the products was measured by the amount of cDNA amplified and using amplification reactions of a 359-bp fragment from 18S cDNA as the control. The amount was normalized using 18S as a standard. Primers used for detection are shown in Table I.

### Flow cytometry

Subsequent to injection of MSCs, the rats were sacrificed at day 7 and 14. Lungs were removed and enzymatically digested using dispase (BD Biosciences, San Jose, CA, USA) and collagenases I (Sigma, St. Louis, MO, USA), yielding a mixed population of lung cells. The cells were passaged through 70 μm filters to obtain single-cell suspensions and analyzed by flow cytometry using the FACSCalibur flow cytometer. GFP^+^ cells were gated in the green fluorescent channel, counted and recorded as a percentage of nucleated cells. MSCs transfected with adenovirus-GFP were used as a positive control, while non-injected MSC lung cells were used as a negative control. Data analysis for all flow cytometry experiments was performed using FloJo software (Tree Star, Inc., Ashland, OR, USA).

### Histologic examination

The left lung was inflated with 4% paraformaldehyde through the trachea for 16 h followed by paraffin-embedding. Sections (5 μm) were cut for hematoxylin and eosin (H&E) and Masson’s trichrome staining. H&E staining was performed according to the manufacturer’s instructions. The sections were stained with H&E to determine histologic structure integrity. The severity of pulmonary fibrosis in lung sections stained for collagen with Masson’s trichrome stains was determined by the histopathologist, who was blinded to the protocol design.

### Detection of MSC differentiation in vitro

To assess the regulation of Wnt/β-catenin signaling on the differentiation of MSCs, 100 ng/ml Wnt3α and 20 ng/ml DKK1 (Peprotech, Inc., Rocky Hill, NJ, USA) were added into the cultured MSCs. MSCs were treated with Wnt3 and DKK1 for 14 days, and then MSCs were detected by immunofluorescence analysis as previously described (25). The primary antibodies were employed as follows: rabbit anti-β-catenin, rabbit anti-α-SMA and mouse anti-vimentin (all antibodies purchased from Abcam, Inc.).

### Western blotting

Cell proteins were obtained from MSCs and tissue proteins were obtained from the right lower lung in each group. Western blot analysis of cellular lysates was performed as previously described (25). Briefly, cells or tissues were lysed in ice-cold extraction buffer containing protease inhibitor cocktail (Roche Applied Science, Indianapolis, IN, USA) for 30 min. The whole lysates were then centrifuged at 12,000 × g for 30 min, and the protein concentration in the supernatant was determined using BCA assays. Proteins were separated using 12% SDS-polyacrylamide gel electrophoresis and were electrophoretically transferred to polyvinylidene fluoride (PVDF) membranes using standard procedures. The membranes were incubated at 37ºC for 1 h in blocking buffer (PBS, 0.1% Tween-20, 1% BSA and 5% non-fat milk). The primary antibodies were added to the membranes and incubated at 4ºC for 16 h. After three washes in PBS, the membranes were incubated with the secondary antibody (horseradish peroxidase-conjugated goat anti-rabbit/mouse IgG; Boster Biological Technology Ltd., Wuhan, Hubei, China) at 37ºC for 1 h. Immunoreactive protein bands were detected using an Odyssey Scanning System (LI-COR Biosciences, Lincoln, NE, USA). The primary antibodies employed were: rabbit anti-CXCR4, rabbit anti-SDF-1, rabbit anti-β-catenin, rabbit anti-MMP-2, rabbit anti-α-SMA, mouse anti-vimentin and mouse anti-β-actin (Abcam, Inc.).

### Immunofluorescence and immunohistochemical staining

Immunofluorescence staining for the detection of engraftment MSC differentiation and immunohistochemistry on paraffin-embedded sections was performed as previously described (27). Briefly, for immunofluorescence staining, the lung tissue samples were fixed in 4% paraformaldehyde in PBS at 4ºC for 4 h followed by overnight immersion at 4ºC in buffer containing 30% sucrose. The specimens were then embedded in optimal cutting temperature (Sakura Finetek USA Inc., Torrance, CA, USA), and stored at −70ºC until use. The tissue was cut transversely at a thickness of 15 μm and the slides were fixed in acetone at 4ºC for 15 min. The slides were then washed twice in PBS for 5 min and blocked by incubation with 3% BSA in PBS/0.3% Triton X-100 for 30 min at room temperature. After draining this solution from the tissue section, the slides were incubated overnight at 4ºC with primary antibodies [1:200; SDF-1, α-SMA, vimentin, cytokeratin 18 (CK18), cytokeratin 19 (CK19) and pro-surfactant protein C (SP-C)]. After three washes with PBS, these slides were incubated with a secondary antibody (goat anti-rabbit Alexa Fluor 594 or goat anti-mouse Alexa Fluor 594; both from Invitrogen Life Technologies) at a 1:400 dilution in 2% BSA for 1 h at 37ºC in the dark. The nuclei were stained with DAPI (5 μg/ml). The images were captured using a laser scanning confocal fluorescence microscope (Olympus). Negative control tissue sections were similarly prepared from each rat except that no primary antibody was added.

For immunohistochemical staining, tissue was fixed in 4% paraformaldehyde in PBS overnight, and then stored in 70% alcohol at 4ºC until processing for paraffin-embedded tissue sectioning. Sections (5 μm) were mounted onto poly-L-lysine- coated slides, dewaxed in xylene, and rehydrated by sequential rinses in 100, 95, 70 and 50% ethanol. Endogenous peroxidase activity was inhibited using 3% H_2_O_2_ solution for 10 min. After three washes in PBS, non-specific binding sites were blocked with 3% BSA for 30 min at 37ºC, and then incubated overnight at 4ºC with primary antibodies (1:200; α-SMA, vimentin, collagen I, CK18 and CK19). Negative control tissue sections were incubated with IgG isotype replaced primary antibody. A secondary biotinylated anti-mouse or anti-rabbit antibody (1:200; Boster Biological Technology, Ltd.) was added and the slides were incubated for 30 min at 37ºC. After rinsing, the slides were incubated with horseradish peroxidase-conjugated streptavidin and then washed with deionized water. In the subsequent steps, the slides were incubated with diaminobenzidine substrate solution for 10 min, and counterstaining with hematoxylin. Images were captured on a Nikon microscope. A brown reaction product was considered a positive result.

### SDF-1 enzyme-linked immunosorbent assay (ELISA)

The protein concentrations of SDF-1 in the ALI rat serum were measured using ELISA (R&D System, Minneapolis, MN, USA), according to the manufacturer’s instructions. The serum was obtained from the abdominal vein of ALI rats. Briefly, plates were blocked and incubated at room temperature for 1 h, then samples were added (100 μl/well) in duplicate for incubation at 37ºC for 90 min. Biotinylated antibodies were subsequently added (100 μl/well) and incubated at 37ºC for 1 h. Incubation with streptavidin-horseradish-peroxidase (at 37ºC for 30 min) was followed by detection with tetramethylbenzidine (TMB) color developing agent at 37ºC for 30 min. The reaction was stopped by the addition of TMB stop solution. Plates were read on a microplate reader (Alisei Quality System, Italy) using a wavelength of 450 nm.

### Statistical analysis

Experimental results were expressed as mean ± standard deviation (SD). Statistical analyses were performed using one-way ANOVA techniques in Microsoft Excel 2003 using SPSS software. This step was followed by a Student Newman-Keuls’ post-hoc test. P<0.05 was considered to be statistically significant.

## Results

### Characterization of MSCs

**Figure 1:**
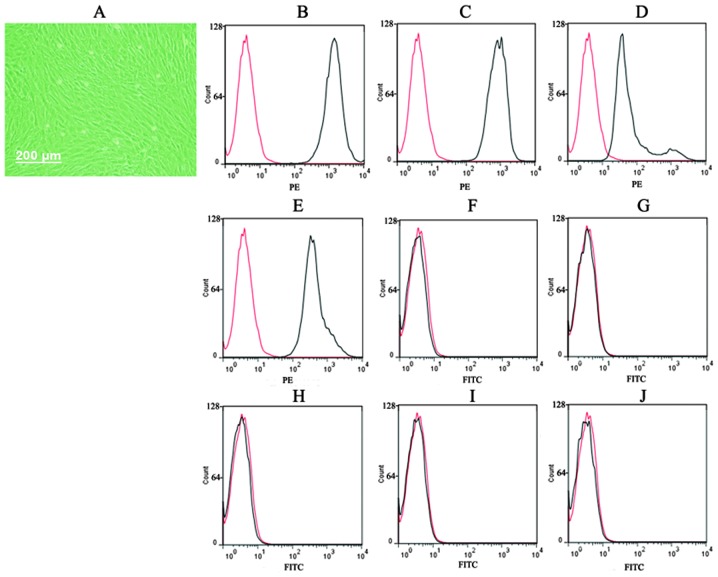
Morphology and flow cytometric analysis of the passage-3 mesenchymal stem cells (MSCs). (A) Image of typical fibroblast-like spindle appearance of MSCs. (B–J) Flow cytometric analysis: MSCs expressed (B–E) CD29, CD44, CD73 and CD90, respectively, but not (F–J) CD11B, CD34, CD45, CD79 and CD133, respectively.

After three passages, the adherent cells showed a typical fibroblast-like and spindle-shaped morphology (Fig. 1A). Results of the FACS analysis demonstrated that cultured MSCs expressed CD29, CD44, CD73 and CD90 (Fig. 1B–E), but not CD11B, CD34, CD45 and CD133 (Fig. 1F–J), indicating that cultured adherent cells were MSCs with high purity. The pure MSCs were used in subsequent experiments.

### Detection of CXCR4 and SDF-1 expression

**Figure 2:**
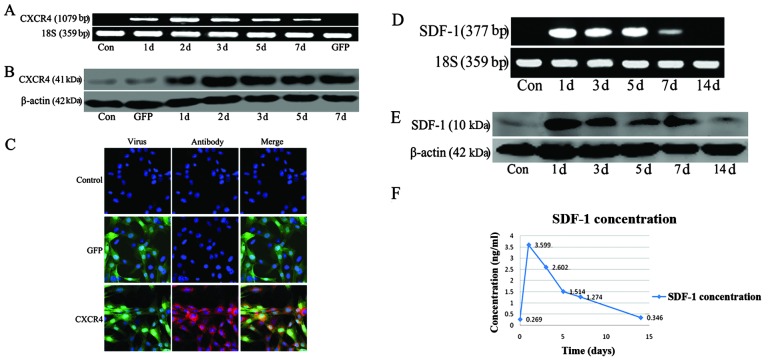
Detection of CXC chemokine receptor (CXCR)4 and stromal cell-derived factor-1 (SDF-1) expression. (A–C) The expression of CXCR4 in transfected mesenchymal stem cells (MSCs). The level of CXCR4 expression in the MSC-Ad-CXCR4 group increased significantly and reached a peak at day 2 after transfection compared with the MSC-GFP reporter gene (Ad-GFP) group and non-transfected MSCs. (A) Reverse transcriptase-polymerase chain reaction (RT-PCR) analysis of CXCR4 gene expression in transfected MSCs. (B) Western blotting detection of CXCR4 protein expression in transfected MSCs. β-actin is used as an inner control. (C) Immunofluorescent analysis of CXCR4 expression in transfected MSCs (green). CXCR4 (red) was stained with goat anti-rabbit Alexa Fluor 594-labeled secondary antibody. Nuclear staining was performed using 4′,6-diamidino-2-phenylindole (DAPI). (D–F) SDF-1 expression level was detected in lung tissue. SDF-1 expression was increased and reached a peak at day 1 following HCl-induced lung injury. (D) RT-PCR analysis of SDF-1 in the lung. (E) Western blotting detection of SDF-1 protein expression in the lung. β-actin is used as an inner control. (F) ELISA analysis for detecting SDF-1 concentrations in serum. d, day(s).

Results of previous studies have indicated that primary MSCs expressed CXCR4 at a low level, whereas passaged MSCs were not able to express CXCR4 following culture (28,29). To aid in cell tracking and explore the role of SDF-1/CXCR4 axis, we transfected the adherent MSCs with an adenovirus carrying the CXCR4 cDNA combined with enhanced green fluorescent protein (eGFP) cDNA. Following transfection, mRNA and the protein expression of CXCR4 in MSCs was increased, reaching a peak at day 2 compared with the expression of MSC-Ad-GFP cells and non-transfection MSCs (Fig. 2A and B). Immunofluorescent analysis also indicated that the transfection efficiency was ~90%, and that MSC-Ad-GFP cells and non-transfection MSCs did not express CXCR4 (Fig. 2C).

After lung injury, we detected the expression of SDF-1 in lung tissue and serum. We found that the mRNA and protein expression of SDF-1 in the lung was increased on day 1, 3, 5 and 7 following HCl-induced injury compared to the control group. The SDF-1 expression reached a peak at day 1 after lung injury and retained a high expression for one week in lung tissue (Fig. 2D and E). The expression of SDF-1 in the serum exhibited the same trend (Fig. 2F).

### SDF-1/CXCR4 axis enhances MSC engraftment

**Figure 3:**
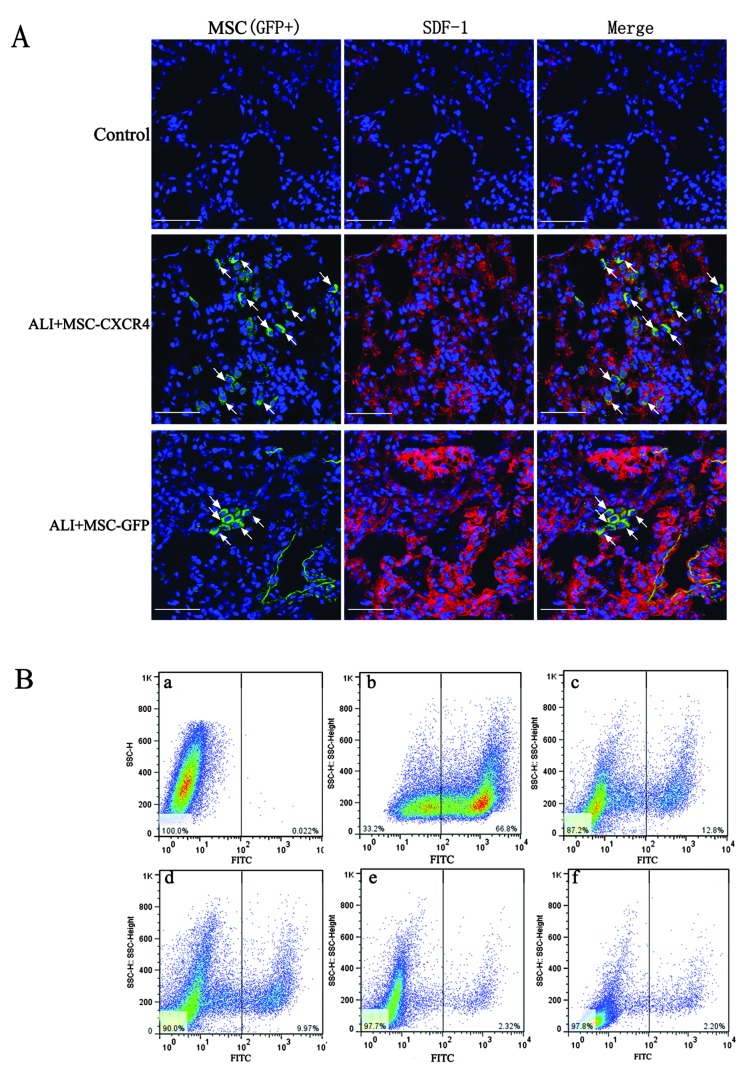
Immunofluorescent staining and flow cytometric analysis was used to detect the engraftment of mesenchymal stem cells (MSCs) (white arrows, green fluorescent cells, GFP^+^ cells) in lung tissue. (A) Immunofluorescent staining detected the engraftment of MSCs. No GFP^+^ cells (MSCs) are detectable in the control lung tissue, while the expression of stromal cell-derived factor (SDF)-1 (red) is very low. GFP^+^ cells are detectable in acute lung injury (ALI)+MSC-CXC chemokine receptor (CXCR)4 and ALI+MSC-GFP groups. More GFP^+^ cells were identified at SDF-1-expressed sites in the ALI+MSC-CXCR4 group compared with the ALI+MSC-GFP group. Scale bar, 50 μm. (B) Flow cytometric analysis for the engraftment ratio of MSCs in lung tissue. MSCs transfected with Ad-CXCR4 or GFP reporter gene (Ad-GFP) were termed GFP^+^ cells. Flow cytometry was used to analyze the GFP^+^ cells in lung cell populations subsequent to MSC administration into lung, as shown in the right area. The SDF-1/CXCR4 axis promotes MSCs to engraft into injured lung tissue. (a) Control group with GFP^+^ cell injection; (b) MSCs transfected with Ad-GFP is used as positive control; (c) MSC-CXCR4 cells injection in ALI lung on day 7 post-transplantation; (d) MSC-GFP cells injection in ALI lung on day 7 post-transplantation; (e) MSC-CXCR4 cells injection in ALI lung on day 14 post-transplantation; (f) MSC-GFP cells injection in ALI lung on day 14 post-transplantation. More GFP^+^ cells were detected in the ALI+MSC-CXCR4 group compared with the ALI+MSC-GFP and control groups.

To assess the engraftment ratio of MSCs at injury sites, we measured the subpopulation of GFP^+^ cells in lung tissue. Immunofluorescence staining was used to detect the engraftment sites of MSCs (GFP^+^ cells) in lung tissue. The results showed that MSCs engrafted the injury sites that expressed SDF-1 at a high level (Fig. 3A). More GFP^+^ cells were identified in SDF-1-expressed sites of the ALI+MSC-CXCR4 group compared to the ALI+MSC-GFP group. Flow cytometry was used to analyze the SDF-1-expressed sites to confirm the role of the SDF-1/CXCR4 axis in the migration of MSCs. Following transplantation of the MSCs for 7 days, ~12% GFP^+^ cells in the cell population were identified in the ALI+MSC-CXCR4 group; however, only ~2% GFP^+^ cells were identified in the ALI+MSC-GFP group (Fig. 3B). The results demonstrated that a high expression of SDF-1 at the injury sites and CXCR4 overexpression in MSCs improved the migration of MSCs into injury sites. These results indicated that the SDF-1/CXCR4 chemotactic axis is crucial in regulating the migration of MSCs. For long-term transplantation, GFP^+^ cells in the cell population showed a similar time-dependent decrease. Following transplantation of the MSCs for 14 days, ~10% GFP^+^ cells were identified at the injury sites in the ALI+MSC-CXCR4 group. Engraftment of MSCs in the lung marked effects on the fibrosis or reparation of injured lung tissue.

### Effects of MSC transplantation in injured lungs

**Figure 4:**
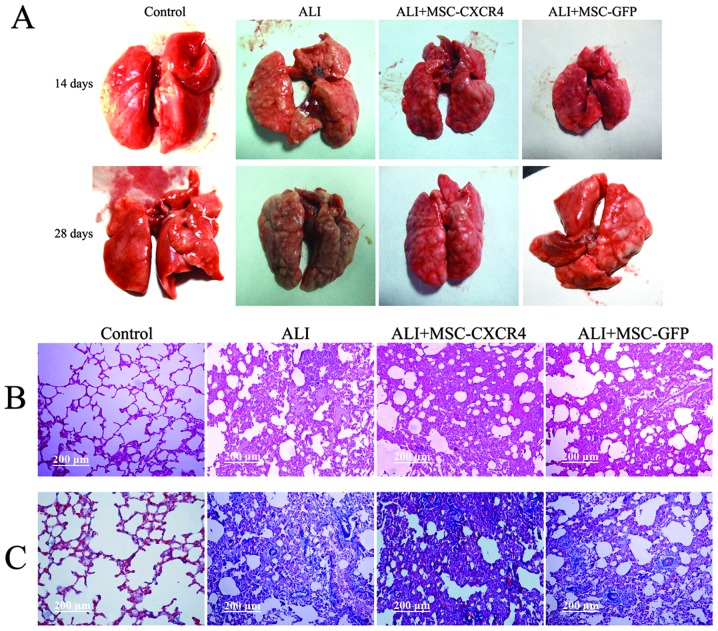
Mesenchymal stem cell (MSC) transplantation did not ameliorate lung injury and pulmonary fibrosis. (A) Representative photographs of whole lungs from all the experimental groups after 14 and 28 days of HCl-induced lung injury. The appearance of lungs in acute lung injury (ALI) group, ALI+MSC-CXC chemokine receptor (CXCR)4 group and ALI+MSC-GFP group were intumescent and fibrotic. They were large and had several scars. Lungs of control group showed a normal aspect. (B) Representative photomicrographs of lung histopathology in all the experimental groups after 28 days of MSC transplantation. Lung sections were stained with hematoxylin-eosin (H&E). MSC transplantation did not reduce lung injury but may aggravate pulmonary fibrosis. Original magnification, ×100. (C) Masson’s trichrome staining analysis of collagen deposition in each group. The presence of interstitial collagen (blue staining) was increased in ALI group and MSC transplantation groups. Original magnification, ×100.

After lung collection, the macroscopic appearance of ALI lungs was intumescent and fibrotic (Fig. 4A). The lung samples were large, yellowish and had several scars when compared with the saline control lungs. However, ALI+MSC-GFP and ALI+MSC-CXCR4 lungs were similar in appearance to that of the ALI lungs. MSC transplantation did not attenuate lung injury and the fibrotic response.

To examine the effect of transplantation of MSCs in ALI rats, serial lung sections obtained from rats following lung injury over 28 days were stained with H&E or Masson’s trichrome staining and examined by light microscopy. Lungs from rats in the ALI group demonstrated marked alteration in lung architecture, with extensive cellular thickening and fibrosis (Fig. 4B). At 28 days after MSC transplantation, H&E staining indicated that the alveolar walls were thickened and pulmonary interstitial fibrosis occurred. Additionally, the extent of fibrosis was as severe as that observed in the ALI group (Fig. 4B). Masson’s trichrome staining demonstrated that collagen deposition (blue staining) in the ALI and MSC transplantation groups was increased compared with the control group (Fig. 4C). Histologic examination indicated that MSC transplantation did not attenuate lung injury and pulmonary fibrosis. However, we hypothesized that the engraftment of MSCs contributes to pulmonary fibrosis. In the fibrogenic environment of the injured lung, MSCs may differentiate into fibroblast phenotype cells and contribute to fibrogenesis.

### MSC transplantation reduced the production of inflammatory cytokines

**Figure 5:**
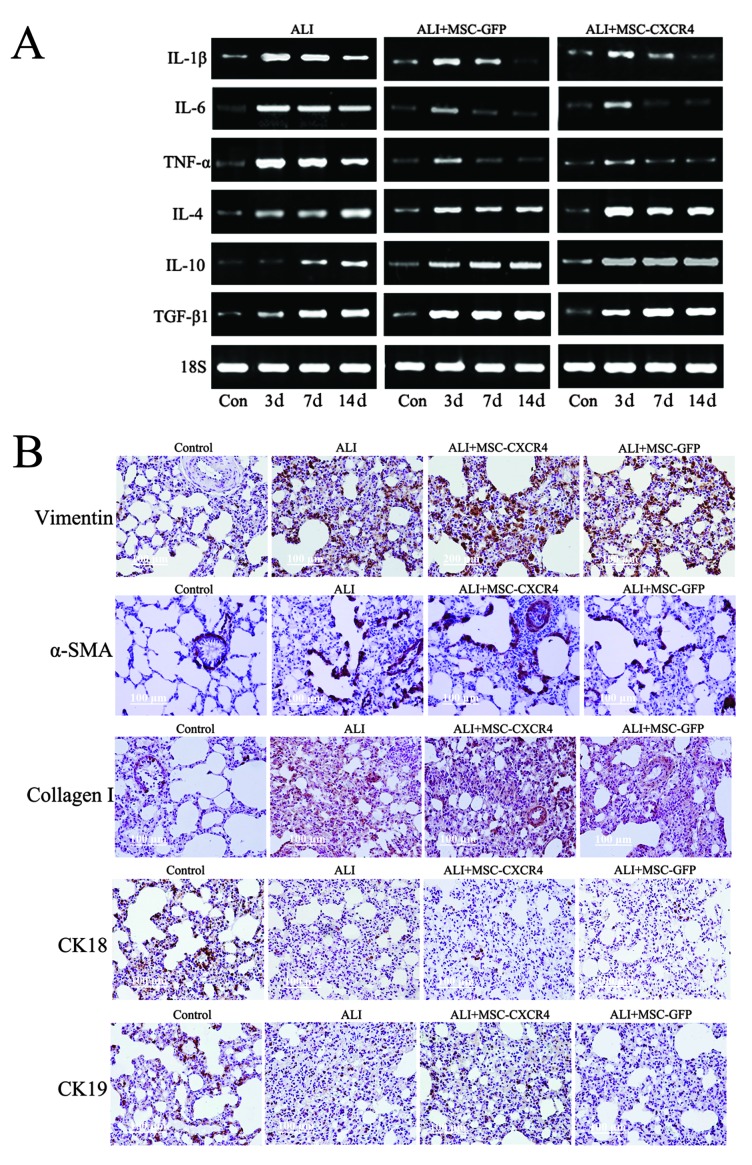
Mesenchymal stem cell (MSC) administration decreased the production of inflammatory cytokines, but did not reduce pulmonary fibrosis and repair injured lung epithelium. (A) Levels of pro-inflammatory cytokines (TNF-α, IL-6 and IL-1β) and anti-inflammatory cytokines (IL-4, IL-10 and TGF-β1) in lung tissue. Transplantation of MSCs reduced the expression levels of TNF-α, IL-6 and IL-1β and increased the expression levels of IL-4 and IL-10 in acute lung injury (ALI)+MSC-CXC chemokine receptor (CXCR)4 and ALI+MSC-GFP groups compared with the ALI group. (B) Immunohistochemical analysis of the expression of fibroblast markers (vimentin, collagen and α-SMA) and epithelial markers (CK18 and CK19) in the lung tissue of all the experiment groups. Transplantation of MSCs did not ameliorate pulmonary fibrosis and repair lung epithelium. The expression of epithelial markers was decreased following transplantation of MSCs, whereas the expression of fibroblast markers was increased. Original magnification, ×200.

To determine the effects of transplantation of MSC on the local inflammatory milieu in the lungs, we determined the expression of several immune system-related cytokines in lung tissue by RT-PCR (Fig. 5A). HCl instillation resulted in increased production of the pro-inflammatory cytokines TNF-α, IL-6 and IL-1β. By contrast, the transplantation of MSCs (ALI+MSC-CXCR4 and ALI+MSC-GFP groups) significantly decreased HCl-induced elevations of TNF-α, IL-6 and IL-1β on 3, 7 and 14 days after ALI, but significantly increased the expression of anti-inflammatory cytokines IL4, IL10 and TGF-β1. The data indicated that MSCs was able to moderate the HCl-induced lung inflammatory response.

### MSC transplantation did not decrease the expression of fibroblast markers

Based on immunohistochemical staining, we detected the expression of fibroblast and epithelial markers in lung tissue (Fig. 5B). After lung injury for 28 days, the expression of α-SMA, vimentin and collagen I was clearly increased, whereas the expression of epithelial markers CK18 and CK19 was decreased in the ALI group compared with the control group. Four weeks after MSC transplantation (ALI+MSC-CXCR4 and ALI+MSC-GFP groups), no beneficial effects on lung injury and pulmonary fibrosis were observed. Additionally, the content of α-SMA, vimentin and collagen I in lung tissue was increased compared with the controls. However, the expression of CK18 and CK19 in lung tissue did not increase after MSC transplantation. The immunohistochemical staining results indicated that MSC transplantation did not reduce pulmonary fibrosis and attenuate lung epithelium injury, but engraftment of MSCs was induced to participate in pulmonary fibrogenesis by some cytokines or signaling pathway at the injury sites.

### Detection of the MSC differentiation in vivo

**Figure 6:**
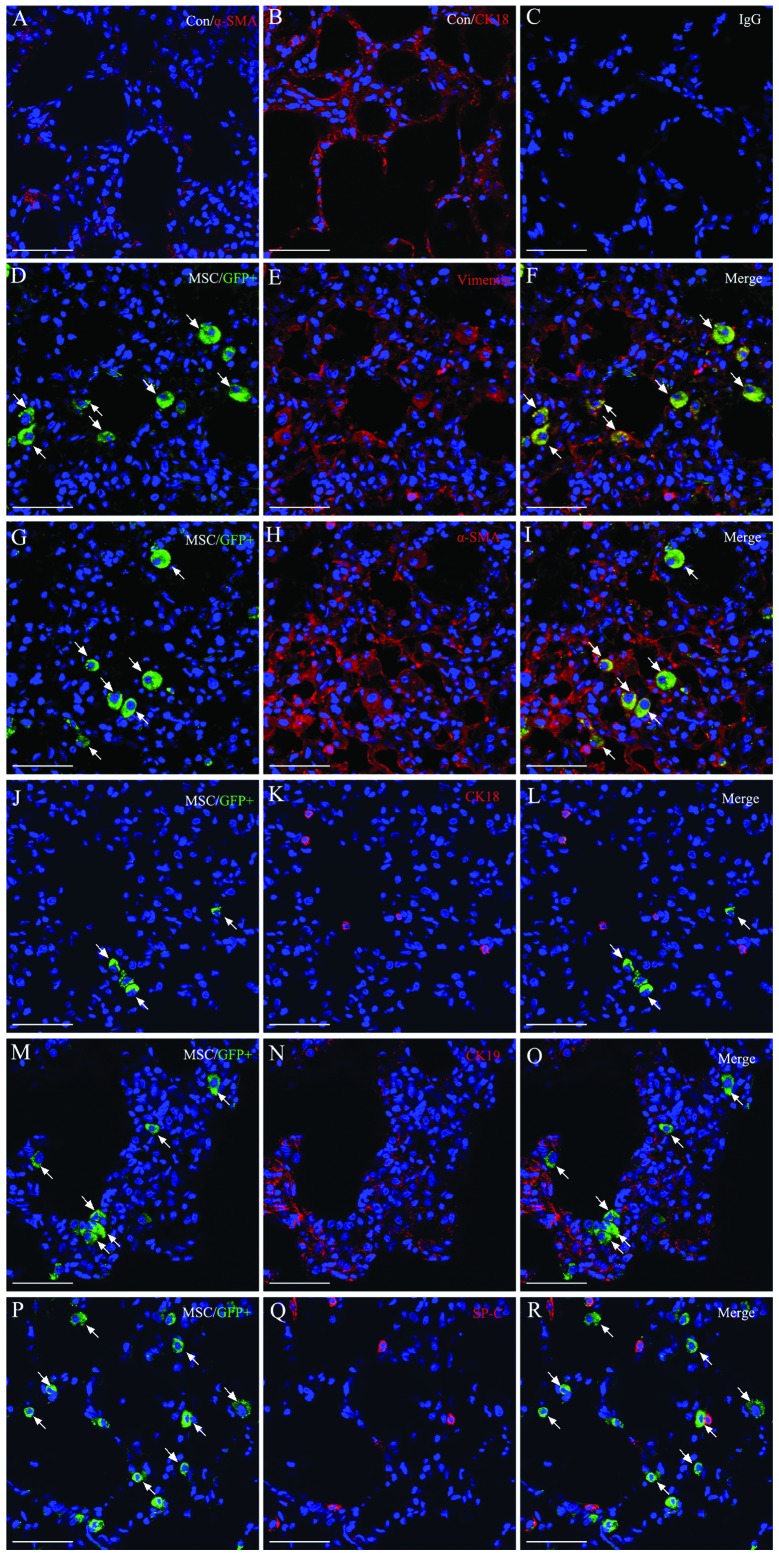
Detection of mesenchymal stem cell (MSC) differentiation 14 days after transplantation in injured lung. Engraftment of MSCs in lung shown as GFP^+^ (green fluorescent cells, white arrows) and antibodies to specific cell-type markers (red); co-localization in each case appears yellow. (A–C) Normal control group for α-SMA, CK18 and IgG. (D–R) Immunofluorescent staining for the engraftment of MSC differentiation in the ALI+MSC-CXC chemokine receptor (CXCR)4 group demonstrated that MSCs expressed myofibroblast or fibroblast markers, but did not express epithelial markers. Engraftment of MSCs was almost differentiated into myofibroblasts or fibroblasts, however, rarely differentiated into lung epithelial cells. Nuclear staining was performed using 4′,6-diamidino-2-phenylindole (DAPI). Scale bar, 50 μm.

Although MSC transplantation decreased inflammatory cytokine production, the beneficial effects of MSCs on lung injury were not observed. We hypothesized that MSC differentiation is important in lung repair or remodeling thereof. To investigate the differentiation of engraftment MSCs *in vivo*, we used immunofluorescent staining to detect the epithelial and fibroblast marker expression of MSCs 14 days after MSCs transplantation in ALI+MSC-CXCR4 group. We found that engraftment of MSCs (GFP^+^ cells show in green) expressed myofibroblast marker α-smooth muscle actin (α-SMA) and fibroblast marker vimentin (Fig. 6A), but rarely expressed epithelial markers CK18, CK19 and SP-C (Fig. 6B). These data suggested that MSCs almost differentiated into lung fibroblasts or myofibroblasts, but did not differentiate into lung epithelial cells. These fibroblasts differentiated from exogenous MSCs may contribute to pulmonary fibrogenesis. Some molecules or signaling pathway may be involved in the regulation of MSCs differentiation at the injury sites.

### Wnt/β-catenin signaling regulates myofibroblast differentiation of MSCs

**Figure 7:**
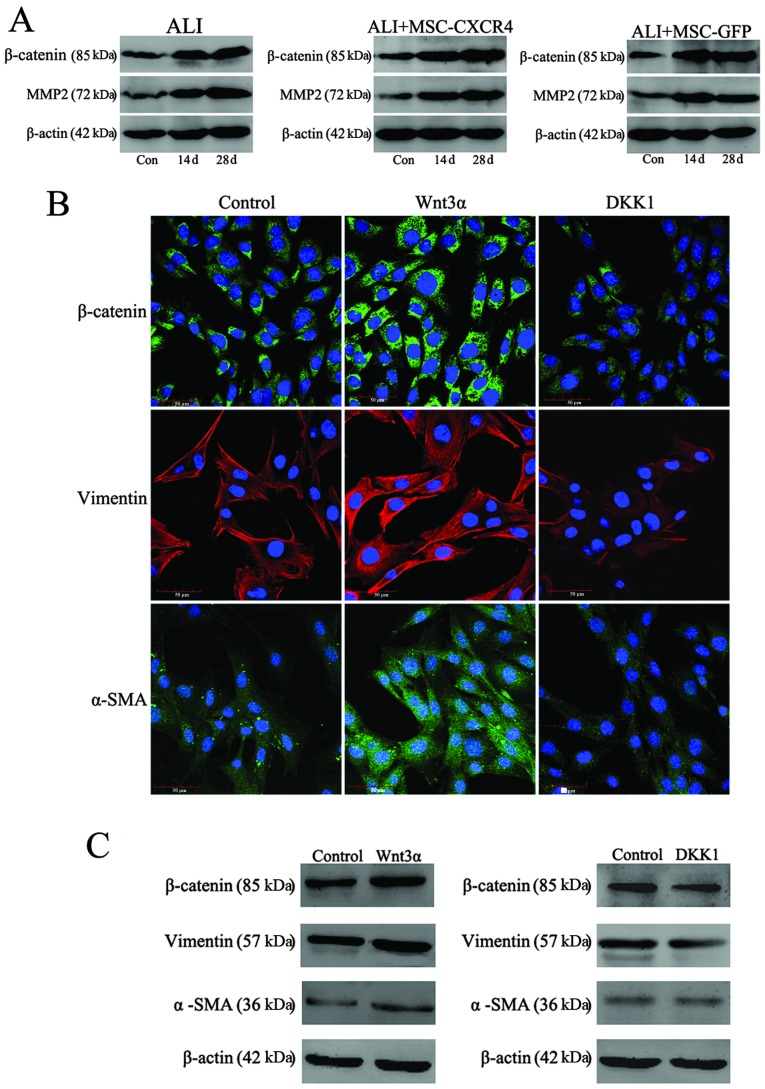
Wnt signaling regulates the differentiation of mesenchymal stem cells (MSCs). (A) The Wnt signaling pathway was highly activated after lung injury. The expression of Wnt signaling components β-catenin and MMP-2 were increased in each group. The activated Wnt signaling may determine the differentiation of MSCs *in vivo*. (B and C) MSCs were cultured until they reached confluence and then treated with Wnt3α and DKK1 for 14 days. Activation of Wnt signaling induces MSCs to differentiate into myofibroblasts which was inhibited by treatment with DKK1. (B) Immunofluorescent analysis of β-catenin, vimentin and α-SMA expression in MSCs. Scale bar, 50 μm. (C) Expression of β-catenin, vimentin and α-SMA was evaluated in whole cell lysates by western blotting. β-actin was used as the control.

Following transplantation, MSCs did not attenuate lung injury and pulmonary fibrosis as we expected, however, MSCs were involved in pulmonary fibrogenesis. We suspect that certain factors or signaling pathway affected the differentiation process of MSCs. It is necessary to explore the regulation mechanisms of MSC differentiation *in vivo*. In a previous study, we demonstrated that Wnt/β-catenin signaling regulates the MSC differentiation *in vitro* (24). In a co-culture system, we demonstrated that activation of Wnt signaling prevented the epithelial differentiation of MSCs, but inhibition of Wnt signaling promoted MSCs to differentiate into epithelial-like cells when MSCs were co-cultured with epithelial cells. Wnt/β-catenin signaling is also involved in regulating lung tissue remodeling, fibrosis or destruction and lung diseases. Therefore, Wnt/β-catenin signaling may regulate the differentiation of MSCs in lung tissue. In this study, we detected the expression of β-catenin and MMP-2, which are the essential components of canonical Wnt signaling. Western blot analysis revealed that the protein expression of β-catenin and MMP-2 was increased significantly in the ALI and ALI+MSC-CXCR4/GFP groups compared with the control group, which demonstrated that Wnt signaling is highly activated (Fig. 7A). It indicated that the abnormal activation of Wnt/β-catenin signaling may induce engraftment of MSCs to differentiate into myofibroblasts or fibroblasts and prevent the epithelial differentiation of MSCs, resulting in tissue repair failure and severe pulmonary fibrosis. The functional role of canonical Wnt signaling in MSC differentiation *in vitro* was investigated. Immunofluorescent staining and western blotting results revealed that treatment of MSCs for 14 days with Wnt3α (100 ng/ml) resulted in an increase in the protein expression of β-catenin, fibroblast marker vimentin and myofibroblast marker α-SMA (Fig. 7B and C). Conversely, DKK1 decreased the expression levels of β-catenin, vimentin and α-SMA in MSCs. The activation of Wnt/β-catenin signaling therefore induced by Wnt3α ameliorated the possibility of MSCs to differentiate into myofibroblasts. By contrast, the inhibition of Wnt/β-catenin signaling caused by DKK1 prevented myofibroblast differentiation of MSCs. It indicated that inhibition of the Wnt/β-catenin signaling pathway following MSC transplantation suggests a positive unique therapeutic approach for lung injury or pulmonary fibrosis.

## Discussion

ALI is characterized by acute hypoxemic respiratory failure, neutrophil accumulation in the lungs, interstitial edema, disruption of epithelial and endothelial integrity and eventually pulmonary fibrosis. Although most lung injury may be repaired by locally derived progenitor cells, findings of previous studies suggest that cells derived from bone marrow, especially MSCs, may also repopulate the lung and repair the injured lung tissue. Mesenchymal stem cell-based therapy is currently a promising and novel treatment for lung injury (28,29). The ability of MSCs to engraft in organs remotely from bone marrow suggests that exogenously administered MSCs contribute to the repair of the injured alveolar epithelium after lung injury. This finding is potentially of significant clinical benefit for the regeneration of injured lung tissue. However, key questions remain to be clarified, including the best route of administration, the most favorable timing of cell infusion, the types of cells differentiated from MSCs, whether engraftment or differentiation requires enhancement of recruitment, and the possible mechanism regulating the differentiation of MSCs. The aim of the current study was to investigate the possible involvement mechanism of MSCs in the treatment for ALI.

SDF-1 and its receptor CXCR4 are important mediators of stem cell recruitment following tissue injury (30–32). Due to the therapeutic potential of MSCs, the SDF-1/CXCR4 axis was used to improve MSC transplantation levels at the injury sites. The results of the present study show that SDF-1 levels in lung tissue and circulating blood increased after HCl-induced lung injury, a finding that is consistent with previous studies (18,33). After transfected MSC administration, using flow cytometric and immunofluorescent analyses, we confirmed that transplantation of MSCs overexpressing CXCR4 specifically traffic to the injured lung which was induced by the gradient SDF-1. The number of engraftment MSCs in the ALI+MSC-CXCR4 group was more than that in the ALI+MSC-GFP and control groups. Therefore, a high expression of SDF-1 at injury sites and overexpressed CXCR4 on MSCs promoted more MSCs to engraft into injured lung tissue sites. The SDF-1/CXCR4 axis promotes exogenous MSCs to migrate and engraft into injured lung tissue. Thus MSCs are crucial in lung remodeling. Based on this, we were able to study clearly the mechanisms of injured lung epithelium reparation following MSC transplantation.

Although the SDF-1/CXCR4 axis increased the number of engraftment of MSCs, we found that engraftment of MSCs did not ameliorate lung injury or pulmonary fibrosis. The amount of collagen deposition in the MSC transplantation group was not reduced by the administration of MSCs, but was as severe as in the ALI group. The results of immunohistochemical staining indicated that MSC transplantation did not ameliorate the epithelium injury and repair epithelium integrity. However, the results demonstrated that the hallmark of pulmonary fibrosis increased. Compared with the control group, the expression of α-SMA, vimentin and collagen I was increased in the lung tissue after lung injury and MSC transplantation, suggesting pulmonary fibrogenesis. Circulating exogenous MSCs may act as a significant source of lung fibroblasts in response to lung injury. We hypothesized that in the fibrogenic environment of the injured lung, the engraftment of MSCs did not differentiate into lung epithelial cells, but may assume a fibroblast phenotype and contribute to fibrogenesis. Immunofluorescence results demonstrated that the engraftment of MSCs rarely differentiated into lung epithelial cells, but almost differentiated into myofibroblasts which contribute to pulmonary fibrogenesis. This result may explain the reason for engraftment of MSCs not ameliorating lung injury and fibrosis.

Recent studies have also shown that bone marrow-derived circulating progenitor cells, including MSCs, accumulate in the lung and contribute to pulmonary fibrosis after lung injury (18,34–36). In other studies, however, a protective rather than a pro-fibrotic effect of bone marrow-derived MSCs has been reported (17,37–39). These contradictory data indicate that the role of MSCs in the reparation or pathogenesis of pulmonary fibrosis needs to be clarified. The differentiation process of MSCs may be regulated by some cytokines and specific signaling pathways at the injury sites. Although we found that MSC transplantation decreased the pulmonary inflammatory process, the pulmonary fibrosis was not completely prevented. This may be because some of the fibrotic response signaling is due to activation of fibroblasts already present in the lungs. Some possible signaling pathways in injured lung may regulate the differentiation of the exogenous MSCs, however, this should be further investigated.

The Wnt/β-catenin signaling has been shown to be a crucial mechanism in regulating embryonic development, cell proliferation and motility, and cell fate determination (40,41). Wnt signaling is involved in the regulation of the differentiation process of MSCs, including adipogenesis, osteogenesis and myogenesis. Previously, we demonstrated that Wnt signaling affected the epithelial differentiation process of MSCs in a co-culture system (24). Wnt signaling pathway is also involved in the regulation of tissue homeostasis, tissue damage and remodeling, injury termination, tissue repair or destruction and tissue diseases (42,43). Mounting evidence has suggested that aberrant activation of Wnt signaling linked to the pathogenesis of fibrotic lung disease, chronic obstructive pulmonary disease, bleomycin-induced idiopathic pulmonary fibrosis and dysregulated wound-healing response (20,44). Findings of those studies suggested that Wnt/β-catenin signaling is relevant to the pathogenesis of pulmonary fibrosis. In the present study, we detected the expression of Wnt signaling in each group. We found that the expression of β-catenin and MMP-2 in the ALI and MSC transplantation groups was upregulated compared with the control group. It indicated that Wnt signaling was highly activated in lung tissue following lung injury. In addition, we have previously demonstrated that the activation of Wnt/β-catenin prevented the epithelial differentiation of MSCs co-cultured with epithelial cells *in vitro*. However, the downregulation of Wnt/β-catenin expression via Wnt antagonists DKK1 promoted MSCs to differentiate into epithelial cells. In the present *in vitro* study, we found that the activation of the Wnt/β-catenin signaling induced the expression of vimentin and α-SMA in MSCs, suggesting that activated Wnt signaling promoted MSCs to differentiate into myofibroblasts. Therefore, Wnt signaling plays a critical role in regulating engraftment of MSC differentiation following transplantation in an HCl-induced lung injury model. The abnormal activated Wnt signaling may promote myofibroblast differentiation of MSCs to aggravate pulmonary fibrosis, however, it prevented epithelial differentiation of MSCs, resulting in lung repair failure. This study suggested that Wnt/β-catenin signaling may act as a control point in the treatment for lung diseases, including ALI and pulmonary fibrosis. Our *in vitro* study has demonstrated that pharmacologic inhibition of the Wnt/β-catenin signaling by DKK1 was able to prevent the myofibroblast differentiation of MSCs. Additionally, aberrant activated Wnt/β-catenin signaling is relevant to the pathogenesis of pulmonary fibrosis via regulation of MSC differentiation after lung injury.

In conclusion, we utilized a rat model of HCl-induced acute lung injury to evaluate the positive effects of MSCs in lung. The results showed that engraftment of MSCs may act as a source of new fibroblasts to promote pulmonary fibrogenesis under lung tissue fibrotic conditions. Our data provides evidence that Wnt/β-catenin signaling plays a critical role in regulating the differentiation of MSCs *in vivo*. Aberrant activated Wnt/β-catenin signaling after lung injury induced the engraftment of MSCs to differentiate into myofibroblasts to contribute to pulmonary fibrogenesis. MSCs have the ability for self-renewal, unlimited proliferation and multi-potential differentiation, making them attractive candidates for tissue repair or malignant change. The possibility of utilizing MSCs as a type of cellular therapy for conditions such as ALI is crucial. Therefore the manner in which *in vivo* environments affect MSCs remains to be determined. Our findings suggest that Wnt signaling is an essential mechanism of regulating MSC differentiation *in vivo*. This study has shown that aberrant activated Wnt/β-catenin signaling is linked to the pathogenesis of pulmonary fibrosis via induction of myofibroblast differentiation of MSCs following lung injury.

## Figures and Tables

**Table I:** Primer sequences for RT-PCR.

Gene name	Forward (5′–3′)	Reverse (5′–3′)
TNF-α	ACGCTCTTCTGTCTACTG	GGATGAACACGCCAGTCG
IL-1β	GAAGTCAAGACCAAAGTGG	TGAAGTCAACTATGTCCCG
IL-6	GAAATGAGAAAAGAGTTGTGC	GGAAGTTGGGGTAGGAAGGAC
IL-4	TCTCACGTCACTGACTGTA	CTTTCAGTGTTGTGAGCGT
IL-10	CACTGCTATGTTGCCTGCTC	TTCATGGCCTTGTAGACACC
TGF-β1	CTTCAGCTCCACAGAGAAGAACTGC	CACGATCATGTTGGACAACTGCTCC
18S	TTTGGTCGCTCGCTCCTC	GCTGCCTTCCTTGGATGTG

RT-PCR, transcription-polymerase chain reaction; TNF-α, tumor necrosis factor-α; TGF-β1, transforming growth factor-β1.
