# [Corrigendum] Activated Wnt signaling induces myofibroblast differentiation of mesenchymal stem cells, contributing to pulmonary fibrosis

**DOI:** 10.3892/ijmm.2026.5867

**Published:** 2026-05-20

**Authors:** Zhaorui Sun, Cong Wang, Chaowen Shi, Fangfang Sun, Xiaomeng Xu, Weiping Qian, Shinan Nie, Xiaodong Han

Int J Mol Med 33: 1097-1109, 2014; DOI: 10.3892/ijmm.2014.1672

Following the publication of the above article, a concerned reader drew to the authors' attention that the immunohistochemical data shown for the 'Collagen I/Control' panel in Fig. 5B on p. 1104, and the 'β-catenin/DKK' panel for the immunofluorescence data shown in Fig. 7B on p. 1106, subsequently appeared in a pair of later publications by the same research group. In addition, in Fig. 5B, the 'Vimentin/ALI' and 'Vimentin/ALI+MSC-GFP' data panels were found to contain an overlapping section, such that data which were intended to show the results of differently performed experiments had apparently been derived from the same original source. Furthermore, upon performing an independent analysis of the data in this paper in the Editorial Office, it also came to light that the 'α-SMA/ALI+MSC-CXCR4' data panel in Fig. 5B had subsequently reappeared in an article by the same research group; the Control panel for the 'MSC (GFP+)' experiment in Fig. 3A on p. 1102 was matching with the Control panel shown in Fig. 6A on p. 1105; certain of the β-actin and MMP2 protein bands shown for the ALI+MSC-CXCR4 and ALI+MSC-GFP experiments in Fig. 7A appeared to be identical in the two sets of western gels; and finally, in Fig. 5A, two sets of data [namely, the data for the IL-6 and TNF-α blots for the ALI+MSC-GFP experiments (central panel of blots), and the pair of Con and 3d 18S blots for the ALI experiments and the 7d and 14d 18S blots for the ALI+MSC-GFP experiments], bore strikingly resemblances to each other.

On re-examining their original data, the authors realized that they had inadvertently included some of the data incorrectly in Figs. 5, 6 and 7. The revised versions of these three figures, now featuring the correct data for Fig. 5A (the PCR analysis results of TNF-α and 18S in the ALI+MSC-GFP group), Fig. 5B (vimentin antibody immunohistochemical staining of the ALI+MSC-GFP group, α-SMA antibody immunohistochemical staining of the ALI+MSC-CXCR4 group, and Collagen I antibody immunohistochemical staining of the Control group); Fig. 6A (α-SMA immunofluorescence staining), Fig. 6C (IgG immunofluorescence staining), Fig. 7A (western blotting results of β-catenin in the ALI group, MMP2 and β-actin in the ALI+MSC-CXCR4 group, and MMP2 and β-actin in the ALI+MSC-GFP group) and Fig. 7B (β-catenin antibody immunofluorescence staining of the DKK1 group), are shown on the subsequent three pages. The image duplications were caused by accidental mix-up of the files during figure sorting and final manuscript preparation. The authors regret that they did not perform more rigorous cross-checking of the figures before submission. The corrected figures are consistent with the original experimental data; moreover, there are now no overlaps with any of the group's previously published work, Notably, the overall experimental results and scientific conclusions of the article remain entirely unchanged following the correction of these figures.

All the authors agree with the publication of this corrigendum, and they are grateful to the Editor of *International Journal of Molecular Medicine* for granting them the opportunity to publish this; furthermore, they apologize to the readership for any inconvenience caused.

## Figures and Tables

**Figure 5:**
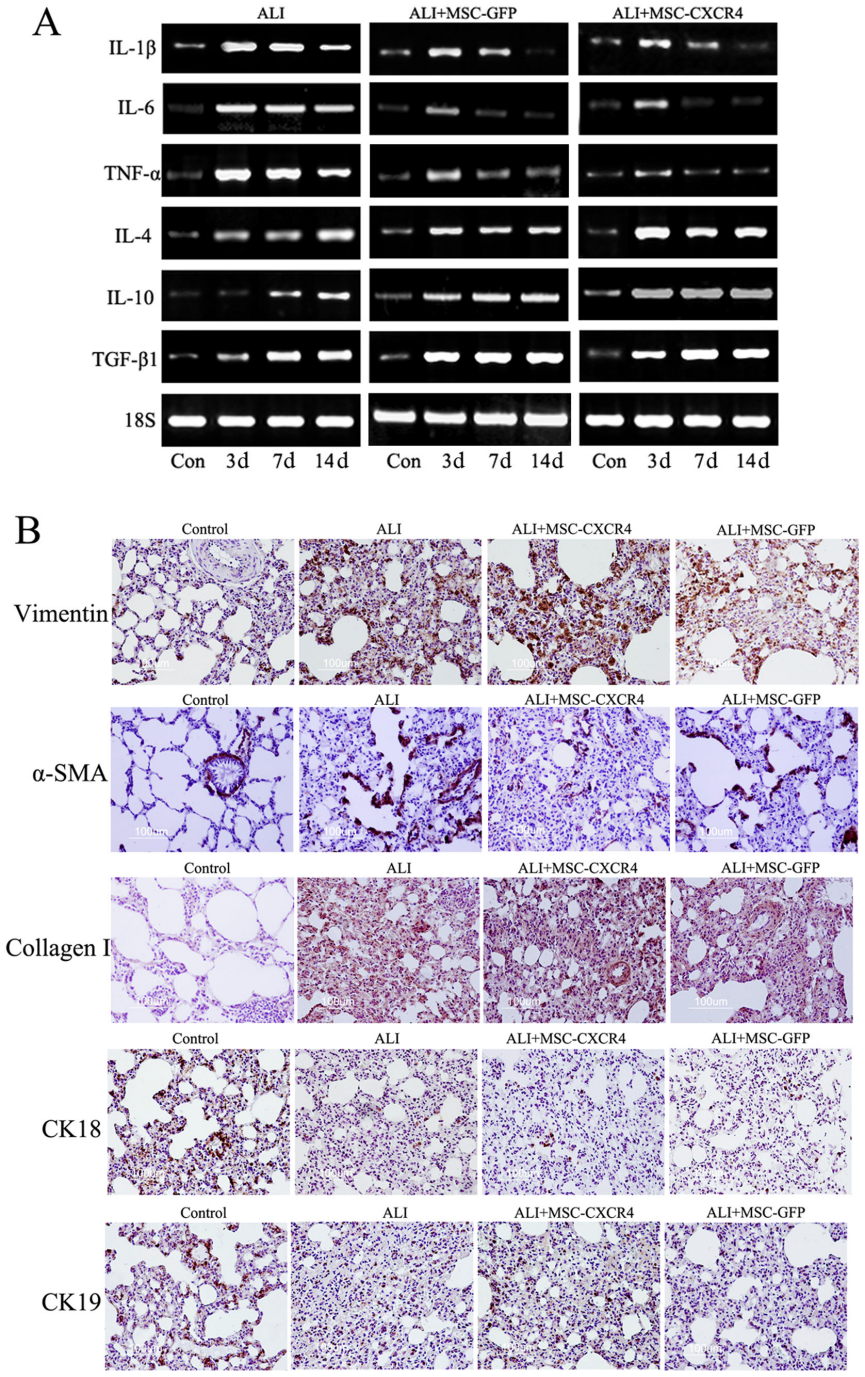
Mesenchymal stem cell (MSC) administration decreased the production of inflammatory cytokines, but did not reduce pulmonary fibrosis and repair injured lung epithelium. (A) Levels of pro-inflammatory cytokines (TNF-α, IL-6 and IL-1β) and anti-inflammatory cytokines (IL-4, IL-10 and TGF-β1) in lung tissue. Transplantation of MSCs reduced the expression levels of TNF-α, IL-6 and IL-1β and increased the expression levels of IL-4 and IL-10 in acute lung injury (ALI)+MSC-CXC chemokine receptor (CXCR)4 and ALI+MSC-GFP groups compared with the ALI group. (B) Immunohistochemical analysis of the expression of fibroblast markers (vimentin, collagen and α-SMA) and epithelial markers (CK18 and CK19) in the lung tissue of all the experiment groups. Transplantation of MSCs did not ameliorate pulmonary fibrosis and repair lung epithelium. The expression of epithelial markers was decreased following transplantation of MSCs, whereas the expression of fibroblast markers was increased. Original magnification, ×200.

**Figure 6:**
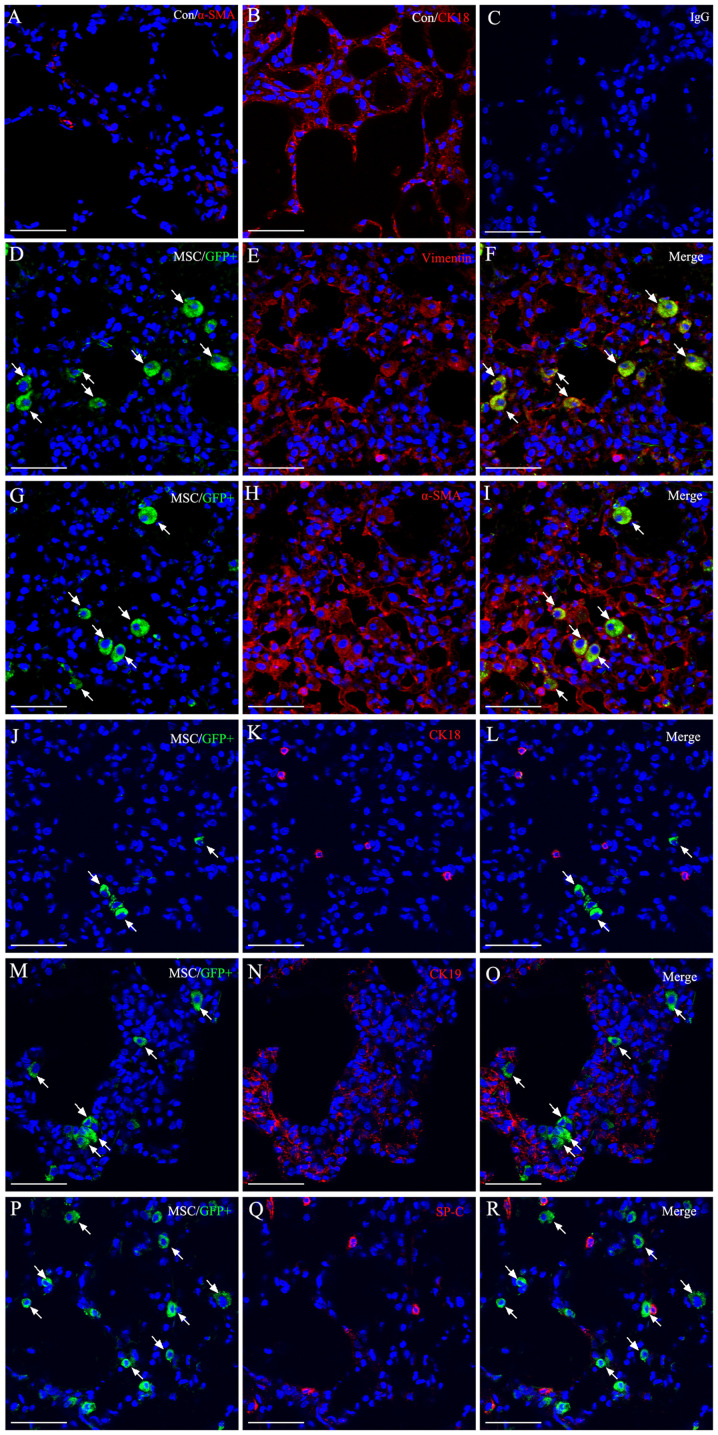
Detection of mesenchymal stem cell (MSC) differentiation 14 days after transplantation in injured lung. Engraftment of MSCs in lung shown as GFP+ (green fluorescent cells, white arrows) and antibodies to specific cell-type markers (red); co-localization in each case appears yellow. (A-C) Normal control group for α-SMA, CK18 and IgG. (D-R) Immunofluorescent staining for the engraftment of MSC differentiation in the ALI+MSC-CXC chemokine receptor (CXCR)4 group demonstrated that MSCs expressed myofibroblast or fibroblast markers, but did not express epithelial markers. Engraftment of MSCs was almost differentiated into myofibroblasts or fibroblasts, however, rarely differentiated into lung epithelial cells. Nuclear staining was performed using 4',6-diamidino-2-phenylindole (DAPI). Scale bar, 50 *μ*m.

**Figure 7:**
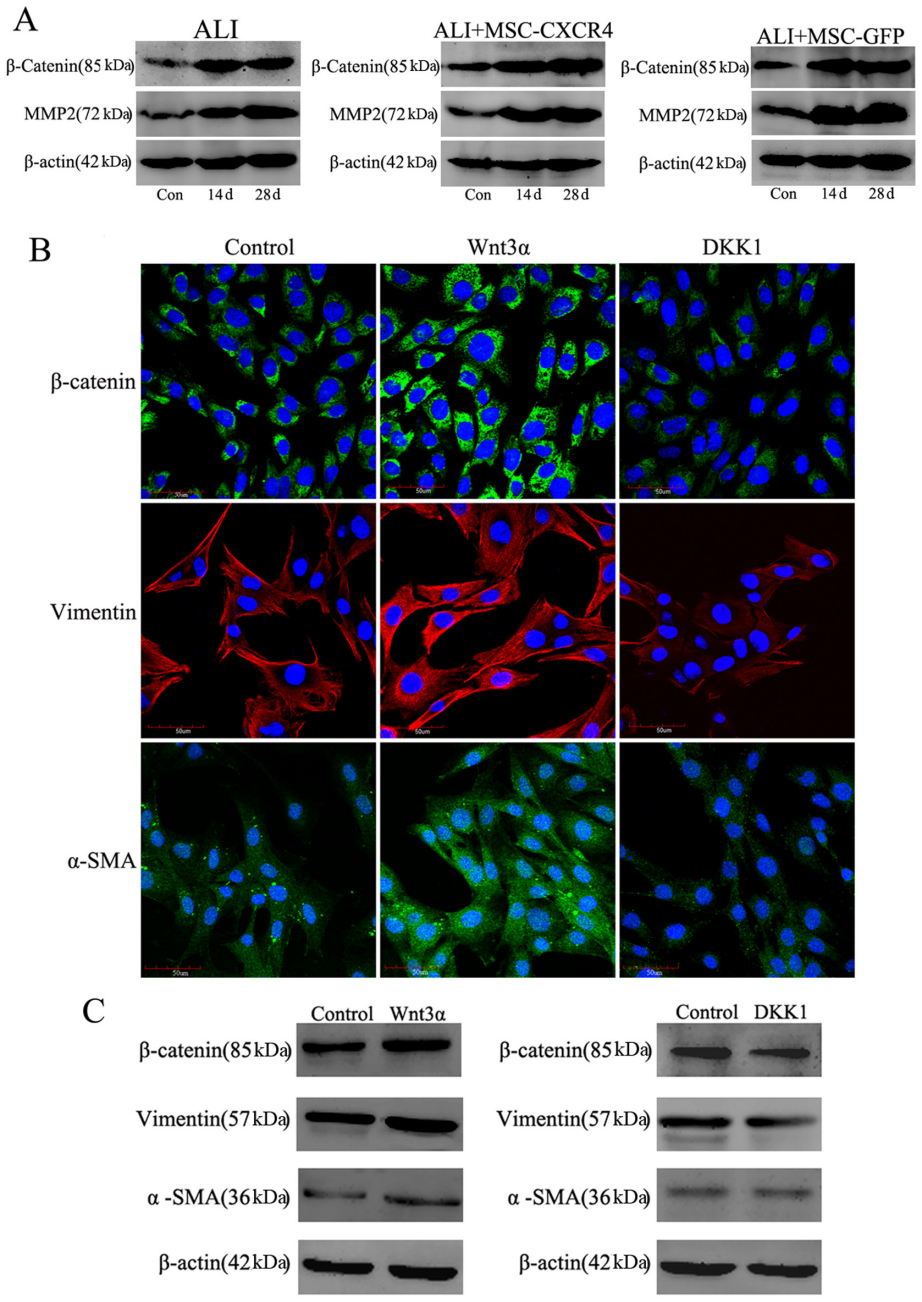
Wnt signaling regulates the differentiation of mesenchymal stem cells (MSCs). (A) The Wnt signaling pathway was highly activated after lung injury. The expression of Wnt signaling components β-catenin and MMP-2 were increased in each group. The activated Wnt signaling may determine the differentiation of MSCs *in vivo*. (B and C) MSCs were cultured until they reached confluence and then treated with Wnt3α and DKK1 for 14 days. Activation of Wnt signaling induces MSCs to differentiate into myofibroblasts which was inhibited by treatment with DKK1. (B) Immunofluorescent analysis of β-catenin, vimentin and α-SMA expression in MSCs. Scale bar, 50 *μ*m. (C) Expression of β-catenin, vimentin and α-SMA was evaluated in whole cell lysates by western blotting. β-actin was used as the control.

